# Editorial: Advances in cardiac imaging and heart failure management

**DOI:** 10.3389/fcvm.2022.1095829

**Published:** 2023-01-09

**Authors:** Giulia Elena Mandoli, Giovanni Benfari, Andrea Baggiano, Roxana Florea, Matteo Cameli

**Affiliations:** ^1^Department of Medical Biotechnologies, Division of Cardiology, University of Siena, Siena, Italy; ^2^Section of Cardiology, Department of Medicine, School of Medicine and Surgery, University of Verona, Verona, Italy; ^3^Peri Operative Cardiology and Cardiovascular Imaging Department, Centro Cardiologico Monzino Istituti di Ricovero e Cura a Carattere Scientifico (IRCCS), Milan, Italy; ^4^Department of Clinical Sciences and Community Health, University of Milan, Milan, Italy; ^5^Emergency Clinical County Hospital, Arad, Romania

**Keywords:** multimodality imaging, heart failure, echocardiography, cardiac CT, cardiac magnetic resonance (CMR), cardiac SPECT

## Introduction

Heart failure is a clinical syndrome with a complex pathophysiology, multiple comorbidities, frequent decompensation, and hospitalizations. It is associated with significant morbidity and mortality, which implies high costs.

This Research Topic presents cardiac imaging novelties in the field of heart failure with the aim of improving management strategies.

## Heart failure with preserved ejection fraction

Heart failure with preserved ejection fraction (HFpEF) is a challenging, incompletely elucidated disease with multiple etiologies and comorbidities. The numerous ongoing studies could help improve the management of this variable population. Cardiovascular imaging, especially echocardiography, plays an important role in this disease, as it represents the first-line imaging modality for diagnosis, stratification, and therapeutic protocols.

The study by Del Torto et al. shows the importance of advanced multimodality cardiovascular imaging (echocardiography, computed tomography—CT, cardiac magnetic resonance—CMR, and nuclear imaging such as scintigraphy and SPECT) for screening and detection of different etiologies in patients with HFpEF. With echocardiography, we assess systolic function and diastolic function of the left ventricle (LV) (mitral flow velocities, mitral annular E' velocity, the E/e' ratio, peak velocity of tricuspid valve regurgitation, and left atrium volume index), as well as additional measures like the velocities in the pulmonary veins and global longitudinal strain (GLS) with speckle tracking echocardiography (STE). CMR is the gold standard for measuring the volume, wall thickness, mass, and ejection fraction for the left and right ventricles. With cardiac CT, on top of coronary anatomy assessment, we can detect valvular calcifications, and assess LV function and the presence and extension of epicardial adipose tissue. Exercise diastolic stress test echocardiography is very important for the assessment of myocardial viability and is recommended to confirm HFpEF in patients with exertional dyspnea. The most studied parameters are mitral E/e' ratio (≥15) and the tricuspid regurgitation velocity (>2.3 m/s), which can indicate an increase in the mean pulmonary capillary wedge pressure (mPCWP) and pulmonary artery systolic pressure (sPAP).

The study by Verwerft et al. presents additional echocardiographic parameters for the exercise diastolic stress test, such as peak exercise septal systolic velocity (exercise S' < 9.5 cm/s), which was the best echocardiographic parameter associated with elevated exercise pulmonary artery wedge pressure (PAWP), and mean pulmonary arterial pressure/cardiac output slope (mPAP/CO ≥ 3.2 mmHg/L). A total of 22 patients were included in this study, among which 14 patients presented a value of exPAWP ≥ 25 mmHg. Although invasive hemodynamic exercise testing with the measurement of PAWP is considered the gold standard to rule in or out patients with HFpEF, it is not used much in daily clinical practice because of the limited expertise and the invasive approach of the method. In addition to this problem, the authors wanted to identify additional non-invasive echocardiographic markers for elevated PAWP because of lacking standardized methods when diagnosing HFpEF non-invasively.

Another possible additional assessment of HFpEF patients is by 3D echocardiography. Wang, Mu et al. introduced a novel echocardiography index based on 3D and tissue Doppler echocardiography for diagnosing and estimating prognosis in HFpEF. This non-invasive index is SVI/S' and is calculated by the ratio between stroke volume indexed by the body surface area (SVI) and tissue Doppler mitral annulus systolic peak velocity (S'). The authors enrolled 104 symptomatic patients with HFpEF who underwent right heart catheterization (RHC). Based on the RHC results, the patients were divided into a HFpEF group (PCWP ≥ 15 mmHg) and non-HFpEF group (PCWP < 15 mmHg) according to the standard cut-off for HFpEF diagnosis. The patients who had a PCWP ≥ 15 mmHg had a low SVI/S' index and showed a poorer prognosis. Also, the non-invasive SVI/S' index was associated with high PCWP measured invasively.

## Acute heart failure

Acute heart failure (AHF) is a life-threatening event that needs a prompt reaction, requiring urgent hospitalization and emergency treatment (1).

The review by Izumo shows the value of transthoracic echocardiography (TTE) in patients with AHF, underlining the fact that it is crucial to determine the etiology. The most used parameter is of course the LV ejection fraction, which reflects the cardiac function and is a valuable diagnostic and prognostic tool. Other relevant parameters are the E/A ratio (a ratio ≥2 indicates elevated LV filling pressures), the E/e' ratio (≥13) by tissue Doppler, the velocity of the tricuspid flow (≥2.8 m/s), and the measurement of the velocity time integral (VTI) in the LVOT tract for the estimation of SV and cardiac output. Also, recent studies have shown that lung ultrasound plays a key role when assessing patients with dyspnea and shock, and that we need to use this tool in addition to TTE.

The study by Mazzola et al. included 86 patients with AHF, 31 cases with and 55 cases without concomitant pneumonia, a common association in the acute setting, especially in older patients. Pneumonia can be both a trigger factor or a following complication of AHF and is associated with high in-hospital mortality. The purpose of this study was to assess B-lines with the help of pulmonary ultrasound using an anterolateral and a posterior approach. The evaluation was realized at admission, after 24, 48 h, and before discharge. Lung ultrasound is non-invasive, rapid, provides high specificity and sensibility, and can even detect residual, subclinical pulmonary congestion at discharge which is proven to predict adverse outcomes. The results showed that only the assessment of the anterolateral B-lines is sufficient for monitoring pulmonary congestion when making a diagnosis, and also for prognostic stratification purposes, because the study showed that they can predict rehospitalization in patients with and without concomitant pneumonia.

Hedwig et al. focus on the assessment of myocardial work, a novel STE-derived parameter, in patients with advanced HF. The study included 105 patients, all evaluated with the help of echocardiography among whom only 94 underwent cardiopulmonary exercise testing. The test was realized on a bicycle ergometer, including the measurement of peak oxygen uptake (peak VO2) and ventilation-carbon dioxide output relation (VE/VCO2) slope, and was stopped only if the patients were exhausted, had angina or significant ECG changes (ST-segment depression), or if the maximum physical capacity was reached. The calculated parameters by STE were the global work index (GWI) and global constructive work (GCW). The results showed that a value of GWI ≤ 455 mmHg% or a GCW ≤ 530 mmHg% was a powerful predictor of outcome in patients with advanced HF.

## Hypertrophic heart

### Hypertrophic cardiomyopathy

Hypertrophic Cardiomyopathy (HCM) is the most common genetic heart disease, often asymptomatic and benign, with mutations in genes encoding sarcomere proteins, characterized by increased wall thickness (maximal WT ≥ 15 mm). The review of Ye et al. focuses on patients with HCM and ventricular arrhythmias, evaluating the role of late gadolinium enhancement (LGE)-CMR. The study included 68 patients with HCM who were grouped into two categories: 31 cases with and 37 cases without demonstrated ventricular arrhythmias. The parameters used for assessment were the LV EF, maximal WT, the diameter of the LA, and %LGE measured by standard volumetric methods. All these parameters were associated with a high development of ventricular arrhythmias events. The authors used a novel marker named *scar entropy* which has been proven to predict adverse cardiac events and all-cause mortality with the possibility to identify arrhythmogenic scars. These findings might be helpful in the future for risk stratification in HCM.

The center of interest for Valdés et al. is LV myocardial strain assessed by CMR imaging techniques. The parameters of LV myocardial strain [global longitudinal (GLS), global radial (GRS), and global circumferential (GCS) strain] were analyzed with the feature tracking (FT) method. The fast strain-encoded (fSENC) method was also used but only for GLS and GCS. The results showed that gender had a strong impact on the strain values derived from FT and fSENC, being more predominant in women than men, while the impact of age on strain is still an inconclusive problem. Another important influence on strain is that of temporal resolution, which is affected by heart rate and the number of cardiac phases. Patients with HCM had lower values of global strain because these values decreased with the increase in cardiac mass, and cut-off values were calculated to help discriminate between healthy and HCM patients.

### Amyloidosis

Amyloidosis is a systemic disease characterized by amyloid fibrils deposition in the extracellular space. Cardiac involvement is the first cause of mortality and morbidity in these patients, and death usually occurs due to electromechanical dissociation or ventricular arrhythmias. There are several types of cardiac amyloidosis, but the most frequent are the primary amyloid light-chain (AL) amyloidosis, and the transthyretin (ATTR) amyloidosis (either variant due to recognized transthyretin gene mutation or wild-type). Many studies have approached this subject, seeking the optimal treatment and management of these patients.

Razvi et al. made a review of current imaging techniques in amyloidosis (echocardiography, CMR, and bone scintigraphy). Echocardiography is the first-line imaging modality for assessment. The typical features are increased LV WT or biventricular implication, thickening of the valves and the interatrial septum, left atrium enlargement, a “speckled” appearance of the myocardium, diastolic dysfunction, and STE “apical sparing”. CMR offers an accurate morphological and functional evaluation, with a detailed myocardial tissue characterization; the latter feature can be obtained without contrast, thanks to the T1 mapping sequence, or after gadolinium-based contrast administration, with LGE sequence (considered the non-invasive gold standard for diagnosis) and with extracellular volume (ECV) estimation. Bone scintigraphy with the use of Tc-99 m pyrophosphate (PYP) has high positive predictive value and specificity, and it is used for additional information in ATTR. All three imaging techniques are useful and important and should be integrated from diagnosis to treatment response monitoring.

### Anderson-Fabry disease

Anderson-Fabry disease is an X-linked lysosomal disorder, caused by a lack or a deficit of the enzyme α-galactosidase A (GLA gene). This disease affects the cardiovascular, renal, and nervous systems, and due to the X-linked transmission, men are usually the most affected. The typical signs and symptoms are anhidrosis/hypohidrosis, angiokeratoma, proteinuria, chronic kidney disease, corneal deposits, and gastrointestinal and cerebrovascular problems. The involvement of the cardiovascular system represents the main cause of death, with the majority being sudden cardiac death events, so it is crucial to make an early diagnosis.

The study by Citro et al. focuses on this disease by selecting patients from daily hospital practices presenting to the echocardiographic laboratory because of LV hypertrophy and “clinical red flags” (classical signs and symptoms). This study was realized in a metropolitan area unexplored before and 30 patients were chosen by specific criteria. Among these patients, three of them (10%) were diagnosed with this disease, and another five were discovered with the help of familiar genetic screening. The interesting finding of this study is that the authors found a completely new mutation gene (mutation c.388A > G (p.Lys130Glu) in exon 3 of the GLA gene) causing a classical phenotype.

### Left ventricle non-compaction

Left Ventricle Non-Compaction (LVNC) is a genetic disorder defined by excessive trabeculations and deep recesses in the left ventricle. This disease is complex, with numerous mutations in genes encoding cardiac sarcomere proteins. It is associated with LV dilatation and systolic dysfunction and sometimes the right ventricle is also involved.

The study by Nemes et al. aims to evaluate by 3D STE the functional and morphological abnormalities of the tricuspid valve annulus in patients with LVNC without right ventricular implication. A total of 21 patients were enrolled, but unfortunately, six of them were excluded because of inferior image quality. The 15 remaining patients were evaluated by a complete and standard 2D echocardiography (LV dimensions, volumes and ejection fraction, left atrial diameter, diastolic function by measurement of transmitral E and A waves, tricuspid annulus plane systolic excursion—TAPSE—and right ventricular fractional area change) and 3D STE. Their results were compared with age and gender-matched healthy patients with negative ECG and echocardiographic findings and with the absence of any disorders. End-systolic and end-diastolic dimensions of the tricuspid annulus were evaluated in 3D echocardiography for the assessment of functional parameters [such as tricuspid annular fractional shortening (TAFS) and tricuspid annular fractional area change (TAFAC)] and morphological parameters (tricuspid annular diameter, tricuspid annular area, and tricuspid annular perimeter—the last two being measured by planimetry). The results showed a significantly dilated end-systolic and end-diastolic tricuspid annulus diameter and area, but with preserved sphincter-like function (TAFAC and TAFS). TAPSE had only a mild correlation with the functional parameters (TAFAC and TAFS).

Another study performed by Wang, Chen et al. shows the influence of right ventricular dysfunction on patients with LVNC. A total of 117 patients were included, 53 patients with RV dysfunction, and 64 patients without RV dysfunction. The criteria for dysfunction included the following parameters: TAPSE < 17 mm, tricuspid S' velocity < 10 cm/s, and RVFAC < 35%. The patients were followed up for a period of more than 5 years and the results showed that the group of patients with RV dysfunction and with an impaired RV global longitudinal strain had a higher risk of all-cause mortality.

## Heart valve disease

### Aortic stenosis

Aortic stenosis (AS) is the second most common valvular heart disease (VHD) with pathological and clinical implications, and is frequently associated with other VHD.

The focal point of the review article by Mantovani et al. is on the latter issue. There is a common association between AS and mitral regurgitation (MR) (20–80%); thus, it is crucial to carefully assess the mechanism of MR for the decision of simultaneous surgical management. When assessing the valves, the study observed that vena contracta is a very reliable parameter due to its independence from afterload, and that the effective regurgitant orifice (ERO) predicts HF and correlates with mortality. In patients undergoing transcatheter aortic valve replacement (TAVI) for severe AS, the presence of moderate-severe MR is associated with poor outcomes and a high rate of mortality and rehospitalization. After TAVI, the MR can worsen or improve by at least one grade. Another common association is AS and tricuspid regurgitation, with worse outcomes, more severe symptoms, and increased mortality and hospitalization rates. Studies have shown that the correction of AS improved tricuspid regurgitation in approximatively 15–30% of patients. According to the guidelines, intervention should be considered in two conditions: when dilatation of the annular tricuspid is present and when there are signs of right HF. The association between AS and mitral stenosis can have various etiologies, but the most frequent cause is rheumatic, followed by degenerative. When assessing the mitral valve in these subjects, the pressure half-time (PHT) method and the continuity equation are not reliable parameters because they lead to overestimation. Bi-valvular surgery is suggested when the mitral valve area (MVA) is ≤ 1.5 cm^2^, and in patients with a high risk of surgical intervention that are not suitable for balloon valvuloplasty, the trans-catheter mitral valve replacement is a new, safe, and efficacious option. The combination between AS and aortic regurgitation (AR) is frequent in the bicuspid aortic valve in rheumatic heart disease. For this assessment, two parameters are indicated: vena contracta and the ERO calculation. The PHT parameter is not reliable, while, in some cases, planimetry might be helpful. When it comes to managing this type of patient, surgery is the first-line therapy and should be done before dilatation and dysfunction of the LV occur.

### Mitral regurgitation

MR is the most common VHD and is classified into two main categories: primary (organic) and secondary (functional). Many studies have focused on this subject, on different aspects of the disease, and on optimal surgical timing.

The review by Pastore et al. focuses on the evaluation of the primary MR and on the optimization of the surgical intervention timing. It is crucial to assess the mitral valve anatomy with the help of specific parameters [annulus area, the chordae and leaflet length, the mitral-aortic angle, the interventricular septum (IVS) thickness, anterior leaflet-IVS distance, the measure of LVOT, and the presence of calcification] and to elucidate the mechanism of the regurgitation. Assessment of the patient is done with the help of 2D and 3D echocardiography focusing on the function and volumes of the LV. STE is also very useful because it provides key information about the LV and left atrium longitudinal function. Pre-operative assessment should mandatorily include the evaluation of right ventricular function, the degree of tricuspid regurgitation, and the tricuspid annulus measurement. Even though TTE could provide enough information, assessment of the patient with transesophageal echocardiography (TEE) is still required because it gives better insights into the mechanism and offers additional parameters. TEE is also important in post-operative evaluation to ensure that there are no unfavorable changes and to assess the integrity of the repair.

### Tricuspid regurgitation

The tricuspid valve (TV), the so-called “forgotten valve”, has caught wider attention in the last few years because of its unique and complex structure and its prognostic relevance. Numerous studies have focused on this issue, trying to discover new modalities to assess the valve, its pathological implications, and to help us better understand the impact on patients.

Margonato et al. present in their review an update regarding the clinical burden of tricuspid regurgitation (TR) in the context of LV systolic dysfunction, and the potential benefit of early TV intervention in these patients. At present, the appropriate time of treatment is still debated, because different subtypes and stages of TR imply different treatment options (isolated surgery, transcatheter options, and palliative procedures). Moderate TR in patients with LV systolic dysfunction is linked to higher mortality, and the parameters which are usually used are ERO > 0.2 cm^2^, vena contracta > 5 mm, and regurgitant volume > 20 ml. These findings are important for the surgical management of patients, and a more aggressive strategy has been proven to prevent significant long-term progression of functional TR.

## Cardiac chambers

### Left atrium

The left atrium (LA) plays a very important role in left heart physiology, and cardiovascular imaging has improved its evaluation in different clinical settings in recent years. It has three major functions: it works as a pump (it delivers blood for ventricular filling), as a reservoir (collecting pulmonary venous blood), and as a conduit (helping blood passage from the left atrium to the left ventricle).

The study by Carpenito et al. focuses on the central role of LA in patients with HF and provides a contemporary review of this topic. For assessment purposes, the method of choice is the left atrial volume indexed to the body surface area (LAVi) because of its strong prediction in cardiac outcomes and risk stratification. The assessment with M-mode and 2D echocardiography (anteroposterior diameter measurement) has proven to be inaccurate so they are not often used in daily clinical practice. A very important aspect is the dilatation of the LA, which offers a prediction of mortality and hospitalization in patients with HFpEF, especially when it is associated with increased pulmonary pressure. Over LA size, peak atrial longitudinal strain (PALS) by STE is correlated with functional capacity during exertion, is a strong predictor of prognosis, and a severely reduced value (< 12.9%) relates to HF symptoms and adverse cardiac events.

### Left ventricle

The LV is the most assessed chamber in daily clinical practice, with the focus often including LV function and size because they are keystones for cardiac diagnostics and prognosis. Countless studies have focused on this topic with diverse purposes and different available imaging techniques.

The study of Airale et al. focuses on the assessment of LV filling pressures using a novel echocardiographic tool named hemodynamic force (HDF) analysis using STE. The final study was composed of 67 patients who were evaluated with TTE and then underwent RHC. The parameter acquired during catheterization was the pulmonary capillary wedge pressure (PCWP) and the results revealed that 33 patients (49.2%) had increased LV filling pressure (PCWP > 15 mmHg). LA volume (LAVi > 34 ml/m^2^), mitral flow velocities, E/A ratio (>14), mitral annular E' velocity (e' septal < 7 cm/s, e' lateral < 10 cm/s), and TR velocity (>2.8 m/s) were then assessed. HDF analysis was obtained with an off-line analysis and the study focused on the diastolic longitudinal component (DLF) because it is closely linked to the diastolic phase of the cardiac cycle and associated with increased LV filling pressure. Based on the echocardiographic findings and the HDF analysis, the authors assembled a scoring system (PCWP prediction score) which was based on LA enlargement, e' septal, LV ejection fraction, and DLF. The score showed high sensitivity and specificity when applied to the studied cohort.

Nemchyna et al. wrote a review about the parameters of LV mechanics with predictive value in patients undergoing surgical ventricular restoration using STE. One hundred and fifty-eight patients were included and evaluated with TTE before the intervention. LV function was assessed using longitudinal parameters measured with the 18-segment model. The authors showed that the basal longitudinal strain (BLS), the end-systolic diameter of the LV, and the fractional shortening, which are parameters of the basal segments of the LV, were strongly associated with outcomes, even more so than global longitudinal strain. Patients with a less impaired BLS had a superior survival rate. Moreover, a preoperatory preserved segmental longitudinal strain was associated with a better improvement in regional wall motion of the LV after surgery.

### Right heart

The right ventricle (RV) has a complex asymmetric geometry, with trabeculations and a profound understanding of its morphology and function is essential for comprehending pathophysiological mechanisms. RV systolic function has an important role in the prediction of adverse outcomes and must be assessed routinely.

Meng et al. studied RV diastolic dysfunction in 71 patients with chronic thromboembolic pulmonary hypertension who underwent pulmonary endarterectomy. The patients were assessed with echocardiography, RHC, and a 6 min walking test. The results showed that the indexed right atrial area (>8.8 cm^2^/m^2^) and the early diastolic strain rate were accurate indices of RV diastolic dysfunction with high sensitivity and specificity.

## Artificial intelligence

Artificial Intelligence (AI) is a new, innovative application in echocardiography that helps and provides navigation through huge amounts of information, can do several automatic tasks, and even offers new opportunities for research.

Machine learning (ML) and deep learning (DL) are branches of artificial intelligence that can serve as diagnostic tools for physicians and can offer alternative pathways in medical management, such as for HF.

The study by Schuuring et al. showed another interesting feature of AI, the so-called *automated view classification* which helps in standardizing views and measurements. It can offer guidance during training by advising how to move the probe in the correct way to get better images and even recognizes views that are incorrect or with an off-axis acquisition. The most performed task was the assessment and quantification of the LV function because it is a crucial parameter in daily clinical practice. Another important solution that AI offers is in the field of VHD because it helps with the sizing of devices in minimally invasive interventions. It is expected that in the future AI will reduce workload, improve the prediction of major adverse cardiac events (MACE) including mortality, and will be a pillar stone in the educational area.

In conclusion, the Research Topic included several starting points for the optimization of HF study and management, ranging from cardiomyopathies to novel advanced imaging techniques.

## Author contributions

All authors listed have made a substantial, direct, and intellectual contribution to the work and approved it for publication.
